# Systematic Analysis of Fertility Conversion via WGCNA Implicates a Compensatory Regulatory Network in a Reverse Thermosensitive Genic Male Sterility Line of Eggplant (*Solanum melongena* L.)

**DOI:** 10.3390/ijms262210873

**Published:** 2025-11-09

**Authors:** Bing Li, Yongpeng Li, Peng Tian, Jingjing Zhang, Wei Liu, Xiurui Gao, Yanrong Wu

**Affiliations:** 1 Institute of Cash Crops, Hebei Academy of Agriculture and Forestry Sciences, Shijiazhuang 050051, China; lbhbnky@163.com (B.L.); tianpengtdc@163.com (P.T.); 1109jing@163.com (J.Z.); liuwei44856@sina.com (W.L.); shijiagao73@126.com (X.G.); 2 Key Laboratory of Agricultural Water Resources, Hebei Laboratory of Agricultural Water-Saving, Center for Agricultural Resources Research, Institute of Genetics and Developmental Biology, Chinese Academy of Sciences, Shijiazhuang 050022, China; liyongpeng0115@163.com

**Keywords:** eggplant, thermosensitive genic male sterility, fertility conversion, RNA-seq, BSA-seq, WGCNA

## Abstract

Thermosensitive genic male sterility (TGMS) lines are vital for two-line hybrid breeding. However, the molecular mechanism in the reverse TGMS line 05ms in eggplant remains unclear. Weighted gene co-expression network analysis (WGCNA) of RNA-seq data revealed modules correlated with fertility conversion enriched in carbohydrate metabolism, lipid metabolism, and mRNA surveillance pathways. Hub genes within these modules were predominantly associated with sugar-related processes, fatty acid metabolism, and nucleotide processing. BSA-seq defined candidate genomic intervals. Integrated analysis of BSA-seq intervals and transcriptomic data identified a candidate gene, *SmHTH*, with consistently lower expression in 05ms compared to S63. Its homologs exhibited temperature-induced expression, possibly compensating for *SmHTH* deficiency under high temperatures to restore fertility. The homologs co-expressed with three transcription factors are likely intricately linked to this response. We propose a compensatory model demonstrating that low *SmHTH* expression at low temperatures disrupts key metabolic pathways, causing male sterility. Conversely, elevated expression of homologous genes and transcription factors (TFs) at higher temperatures compensates for the loss of *SmHTH* function, thereby restoring fertility. The findings of this research not only deepen the theoretical understanding of plant male sterility mechanisms but also provide valuable resources for developing stress-resilient vegetable varieties through modern breeding techniques.

## 1. Introduction

Eggplant (*Solanum melongena* L.) is a major vegetable crop worldwide. Heterosis, commonly exploited through cross-breeding, can enhance yield, quality, and resistance [1]. Male sterile lines are crucial for hybrid seed production, which primarily utilizes either a three-line or two-line system [2,3]. The three-line system relies on cytoplasmic male sterility (CMS), maintainer, and restorer lines, whereas the two-line system uses environmentally sensitive genic male sterile (EGMS) lines, requiring only a male sterile line and a restorer line [4,5,6]. EGMS lines can regain fertility under permissive environmental conditions, offering greater flexibility in pollen parent selection and hybrid combination potential [7,8].

EGMS resources, including photoperiod-sensitive (PGMS), thermosensitive (TGMS), humidity-sensitive (HGMS), and nitrogen-sensitive (NGMS) genic male sterility, have been extensively utilized in rice two-line breeding, with several key genes cloned [9]. Reverse PGMS (rPGMS) lines are sterile under short days (SD) and fertile under long days (LD), analogous to reverse TGMS (rTGMS) lines, which are sterile at low temperatures and fertile at high temperatures [2]. TGMS lines, particularly, are widely used [10]. For instance, Carbon Starved Anther (CSA), encoding a MYB protein, regulates sugar partitioning during pollen development in rice [11]. CRISPR/Cas9 editing of CSA created photo-sensitive lines [12]. UDP-glucose pyrophosphorylase 1 (Ugp1), essential for fertility, exhibits temperature-sensitive splicing, leading to TGMS in rice [13]. Some EGMS-regulating genes themselves lack environmental sensitivity but affect temperature-responsive targets. For example, Thermosensitive Genic Male Sterile 5 (TMS5), encoding ribonuclease Z (RNase Z), degrades transcripts of UbL40 genes, whose expression is induced by high temperature; tms5 mutants accumulate UbL40 mRNA/protein, causing sterility at high temperature [14]. Unstable critical sterility-inducing temperatures (CSIT) can hinder TGMS application. CRITICAL STERILITY-INDUCING TEMPERATURE 2 (CSIT2), an E3 ligase, modulates CSIT in tms5-based lines via ribosome-associated quality control [15]. TMS10, a leucine-rich repeat receptor-like kinase, regulates tapetal development; while not thermosensitive, its homolog TMS10L is induced at low temperature, restoring fertility in tms10 mutants at low but not high temperature [16]. EGMS has also been reported in vegetables like pepper [17], tomato [2], eggplant [18], and Brassica [19], though research in these species lags behind rice, with genetic loci and molecular mechanisms largely unexplored [2]. In eggplant, RNA-seq of CMS line EP26A and its maintainer revealed alterations in oxidative phosphorylation, carbohydrate, and amino acid metabolism pathways [20]. Phytohormones are also implicated in male sterility [21]. Nevertheless, studies on eggplant rTGMS lines are scarce, and the regulatory mechanisms governing fertility conversion remain unclear.

TGMS lines are crucial for two-line hybrid breeding, yet the molecular mechanisms of rTGMS in eggplant remain largely elusive, limiting its application. In this study, we leveraged an F_2_ population from a cross between the rTGMS line 05ms and its fertile parent S63. We generated a comprehensive transcriptome profile across 21 samples and employed Weighted Gene Co-expression Network Analysis (WGCNA) for a systems-level investigation. By integrating these results with BSA-seq data, we aimed to: (i) identify key modules and hub genes co-regulated with fertility conversion; (ii) screen for core candidate regulators within the BSA-seq candidate intervals; and (iii) uncover the regulatory mechanisms underlying thermosensitive fertility conversion in eggplant. Our work provides crucial insights into the molecular basis of fertility conversion and offers a valuable resource for marker-assisted breeding in eggplant.

## 2. Results

### 2.1. RNA-Seq Data Analysis

**Figure 1:**
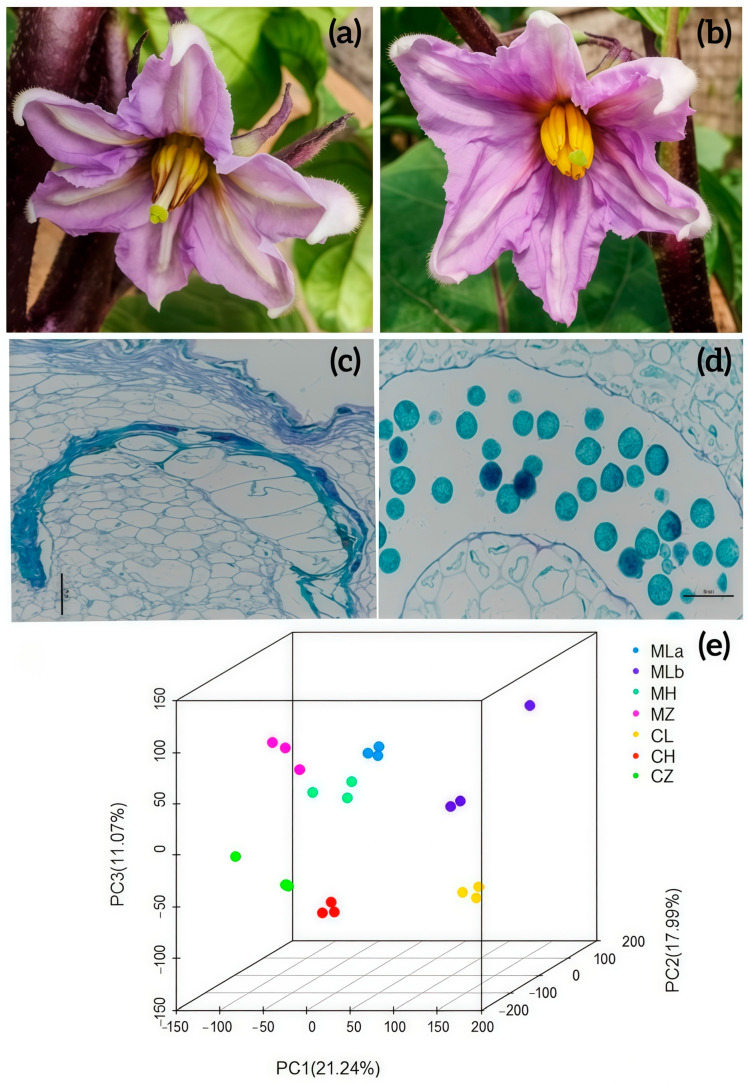
Phenotype comparison of stamens in 05ms and S63 and PCA of all the RNA-seq data. (**a**) The phenotype of stamens in 05ms; (**b**) The phenotype of stamens in S63; (**c**) The cytological comparison of anthers in 05ms; (**d**) The cytological comparison of anthers in S63; (**e**) The PCA result of all the RNA-seq data. (**c**,**d**) 400×, Bar = 100 µm.

To investigate fertility conversion in the rTGMS line 05ms, RNA-seq was performed on buds collected at the anther meiosis stage from 05ms and S63 during spring 2019. Data from this study and previous work [18] were combined for analysis. Samples were designated as follows: for 05ms—ML (sterile, spring low temperature), MH (fertile, summer high temperature), MZ (sterile, autumn low temperature); for S63—CL (spring low temperature), CH (summer high temperature), CZ (autumn low temperature). Sample MLb and CL were from 2019; others from 2016. Details are in Table 1. Fertility differences between 05ms and S63 sown in spring 2019 were evident (Figure 1a,b). Cytological observation showed shriveled anther walls and empty pollen sacs in 05ms compared to S63 (Figure 1c,d), consistent with previous findings. Principal component analysis (PCA) confirmed that replicates clustered together (Figure 1e), supporting the combined analysis.

**Figure 2:**
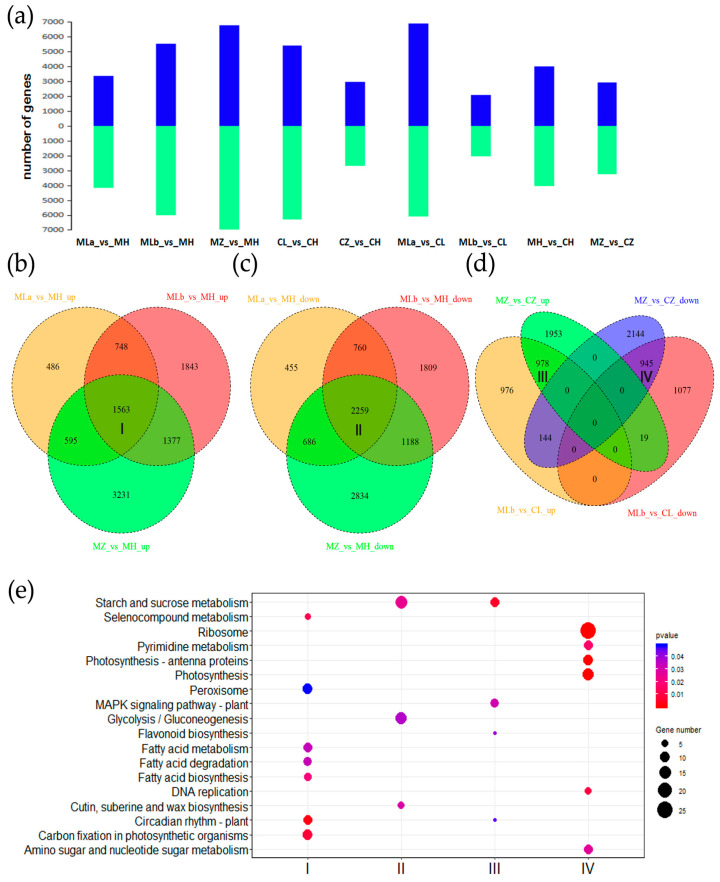
The identification and KEGG enrichment analysis of the differentially expressed genes (DEGs) between different combinations of comparisons between 05ms and S63. (**a**) Number of DEGs in different comparisons; (**b**) Overlapped genes between MLa_vs_MH_up, MLb_vs_MH_up, and MZ_vs_MH_up; (**c**) Overlapped genes between MLa_vs_MH_down, MLb_vs_MH_down, and MZ_vs_MH_down; (**d**) Overlapped genes between MZ_vs_CZ and MLb_vs_CL. (**e**), the KEGG enrichment analysis of gene clusters indicated in (**b**–**d**). I, II: fertility conversion genes in 05ms; III, IV: genotype difference genes between 05ms and S63.

DEGs were identified across nine comparisons: MLa_vs_MH, MLb_vs_MH, MZ_vs_MH, CL_vs_CH, CZ_vs_CH, MLa_vs_CL, MLb_vs_CL, MH_vs_CH, MZ_vs_CZ. The number of DEGs in each comparison is shown in Figure 2a. Environmental conditions had a stronger impact on gene expression than genetic background. For instance, only 4137 DEGs were identified in the MLb_vs_CL comparison, whereas 11,547 and 11,720 DEGs were found in the MLb_vs_MH and CL_vs_CH comparisons, respectively (Figure 2a). To identify genes stably associated with fertility, we analyzed overlapping genes between comparisons. We focused on up-regulated or down-regulated genes shared between 05ms grown at low versus high temperature (Cluster I in Figure 2b and Cluster II in Figure 2c), which may represent key genes for fertility conversion in 05ms. Additionally, overlapping up- or down-regulated genes between MZ_vs_CZ and MLb_vs_CL (Cluster III and IV in Figure 2d), which compare different genotypes under the same low temperature, might explain the fertility difference between 05ms and S63.

KEGG enrichment analysis of these clusters revealed significant involvement of “Starch and sucrose metabolism”, “Glycolysis/Gluconeogenesis”, “Fatty acid biosynthesis”, “Fatty acid degradation”, “Fatty acid metabolism”, “Cutin, suberine and wax biosynthesis”, and “Amino sugar and nucleotide sugar metabolism” (Figure 2e), highlighting roles for carbohydrate and lipid metabolism. GO terms related to sugar or lipid metabolism and cell wall processes (e.g., “cell wall assembly”, “glucan biosynthetic process”) were also enriched (Figures S1A,B and S2A,B), suggesting involvement in anther wall development.

### 2.2. Co-Expression Networks Construction and Key Modules Analysis

**Figure 3:**
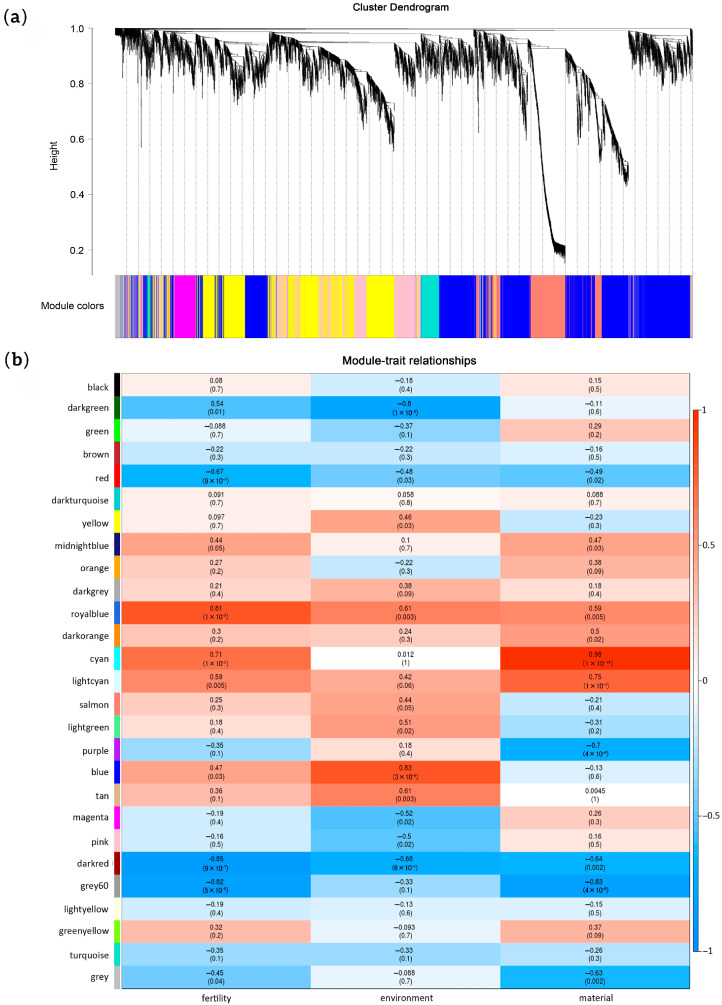
Weighted Gene Co-expression Network Analysis (WGCNA) of gene expression changes in 05ms and S63 under different environments. (**a**) Hierarchical cluster tree showing 27 modules of co-expressed genes; (**b**) Module–trait correlations and corresponding *p*-values (in parentheses).

WGCNA using genes with high expression variance identified 27 modules containing 35 to 3317 genes each (Figure 3a,b). Samples were categorized by fertility (sterile: MLa, MLb, MZ; fertile: MH, CL, CH, CZ), temperature (low: MH, CH; high: MLa, MLb, MZ, CL, CZ), and genotype (05ms: MLa, MLb, MH, MZ; S63: CL, CH, CZ). Module–trait relationship analysis identified modules highly correlated (|r| ≥ 0.8) with these traits: royal blue, dark red, grey60 with fertility conversion; blue, dark green with environment (temperature) response; cyan with genotype (Figure 3b). Expression patterns of genes in these modules were clearly segregated by trait (Figures S3A,C, S4A,C and S5A,C).

GO and KEGG analyses were performed for royal blue, dark red, grey60, blue, dark green, and cyan modules.

**Figure 4:**
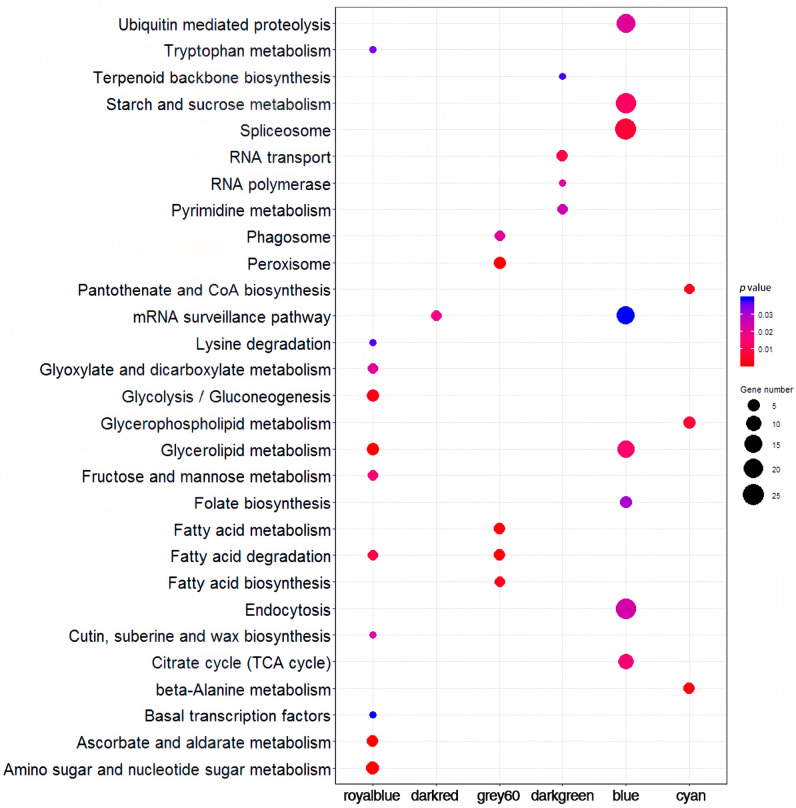
The KEGG enrichment analysis of genes in royal blue, dark red, grey60, dark green, blue, and cyan modules.

- Royal blue (fertility conversion): Enriched KEGG pathways included carbohydrate/lipid metabolism processes (“Glyoxylate/dicarboxylate metabolism”, “Glycolysis/Gluconeogenesis”, “Fructose/mannose metabolism”, “Amino/nucleotide sugar metabolism”, “Glycerolipid metabolism”, “Fatty acid degradation”, “Cutin/suberine/wax biosynthesis”) (Figure 4). GO terms related to glycosyl transfer/glycosylation were enriched (Figure S3B).
- Grey60 (fertility): Enriched KEGG pathways included fatty acid metabolism-related processes (Figure 4). GO terms for hydrolysis and catabolic processes were enriched (Figure S4B).
- Dark red (fertility conversion): The sole enriched KEGG pathway was “mRNA surveillance pathway” (Figure 4). GO terms for RNA activity and tRNA metabolism were enriched (Figure S3D).
- Blue (temperature response): Enriched KEGG pathways related to transcription/translation (Figure 4). GO terms included “cell cycle”, “response to stimulus” (e.g., “heat shock protein binding”), and “transcription factor activity” (Figure S5B).
- Dark green (temperature response): Enriched KEGG pathways related to transcription/translation (Figure 4). GO terms included hydrolysis, ribonucleotide binding, transcription factor complex, and ribosome biogenesis (Figure S5D).
- Cyan (genotype specific): Enriched KEGG pathways were “Pantothenate and CoA biosynthesis”, “Glycerophospholipid metabolism”, “beta-Alanine metabolism” (Figure 4). GO terms related to glucan, cellulose, and oxidoreductase activity were enriched (Figure S4D).

### 2.3. Hub Gene Analysis and Transcription Factors Identification

**Figure 5:**
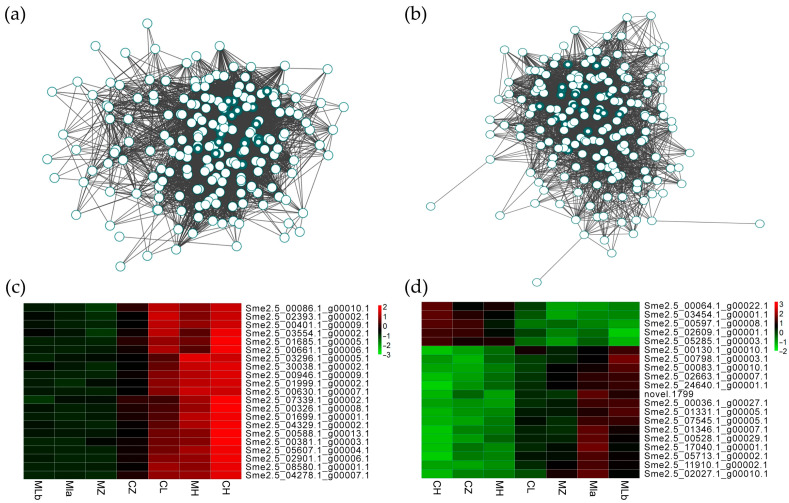
Hub genes in royal blue and dark red modules, circles with heavy color indicating hub genes. (**a**) The visualization of the co-expression network of genes in royal blue; (**b**) The visualization of the co-expression network of genes in dark red. (**c**) Heatmap showing the expression difference of hub genes in royal blue modules in different samples; (**d**) Heatmap showing the expression difference of hub genes in dark red modules in different samples.

Hub genes (top 10% connectivity) were identified [22]. Network visualization for royal blue and dark red modules showed hub genes centrally located (Figure 5a,b). Homology analysis with tomato and Arabidopsis revealed functions for royal blue hub genes: 10/21 were involved in sugar-related processes (SWEET transporters, beta-galactosidases, glycosyltransferases, glycosyl hydrolases, nucleotide sugar transporters). Others were associated with fatty acid metabolism, ABA signaling, GA signaling, or phototropism. Dark red hub genes were involved in nucleotide/RNA processes (tyrosyl-tRNA synthetase, RNA helicase, DNA polymerase, DNA repair enzyme, tRNA isopentenyl transferase, splicing factor), consistent with mRNA surveillance. Hub genes for every module are listed in Tables S1–S6. qRT-PCR confirmed the expression trends of nine selected hub genes, consistent with RNA-seq data (Figure S6).

**Figure 6:**
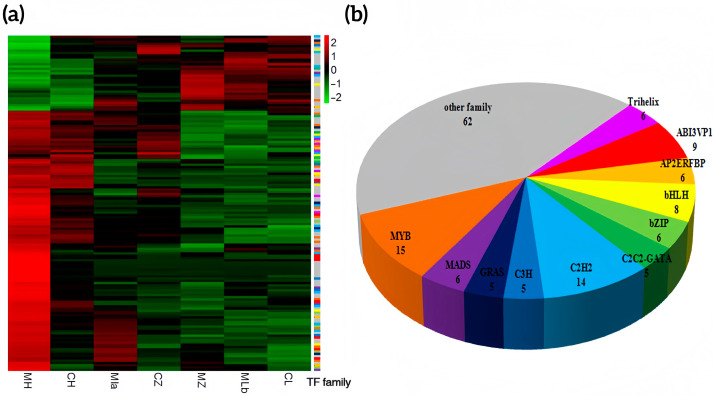
Transcription factors in blue modules. (**a**) Heatmap showing the expression change of transcription factors in the blue module; (**b**) Pie chart showing the family distribution of transcription factors in the blue module.

Given the enrichment of “transcription factor activity, sequence-specific DNA binding” in the blue module, we analyzed TFs therein. Among 86,713 annotated genes, 921 were TFs. The blue module (2919 genes) contained 147 TFs, a significantly higher proportion than the genome background (*p* = 2.31 × 10^−84^), indicating TF sensitivity to environmental changes. Most showed stable expression differences between high and low temperatures (Figure 6a). Predominant TF families included MYB, C2H2, ABI3VP1, bHLH, bZIP, and MADS (Figure 6b).

### 2.4. BSA-Seq Data Analysis

BSA-seq previously identified 26 candidate genes based on sequence variants between 05ms and S63 [18] and 30 scaffolds. As the rTGMS trait in 05ms arose spontaneously from S63, epigenetic changes affecting gene expression could also be causal. Given that the rTGMS phenotype in 05ms originated from a spontaneous mutation in the S63 line and was subsequently stabilized through multiple generations of breeding and selection over 15 years, the observed sequence variations serve as reliable molecular markers for mapping candidate regions. Therefore, we relaxed the screening criteria. To account for potential quantitative trait loci (QTLs) with minor effects, candidate SNPs and InDels were screened genome-wide. For genomic sites where the reference parent and offspring exhibited the same phenotype, loci with an allele index (All-index) below 0.2 in the offspring pool were considered candidate intervals. Conversely, for sites where the reference parent and offspring displayed opposite phenotypes, loci with an All-index greater than 0.8 in the offspring pool were selected as candidate intervals. Based on the established criteria, a total of 159 divergent scaffolds were screened as candidate intervals (Table S7). The 159 candidate scaffolds harbored 1573 genes. After excluding genes with zero expression across all samples, 1157 genes remained for subsequent analysis.

### 2.5. Integrated BSA-Seq and RNA-Seq Analysis

**Figure 7:**
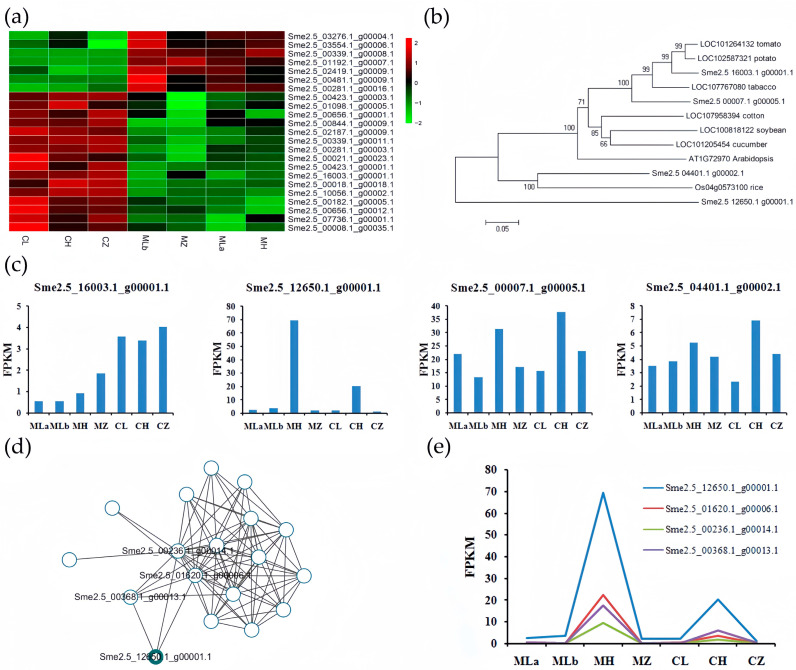
Analysis of the candidate gene and its homolog. (**a**) heatmap showing the expression change in candidate genes selected by BSA-seq; (**b**) Protein homology alignment of *Sme2.5_16003.1_g00001.1* and its homolog in different species; (**c**) The expression change in *Sme2.5_16003.1_g00001.1* and its homologous genes in eggplant; (**d**) The co-expression network of *Sme2.5_12650.1_g00001.1* and transcription factors in blue module using 0.3 as threshold; (**e**) The expression change in *Sme2.5_12650.1_g00001.1* and its co-expressed transcription factor.

To identify the most promising candidate genes within the BSA-seq, we performed an integrated analysis of genomic and transcriptomic data. We first extracted the expression data of 1157 genes located within the 159 candidate scaffolds from our RNA-seq dataset. Following k-means clustering (k = 6) of all 1157 genes, we selected candidate genes from every cluster that showed the clearest expression divergence between 05ms (MLa, MLb, MH, MZ) and S63 (CL, CH, CZ). This practical filtering strategy yielded a final list of 23 candidate genes with reliable functional annotations for further characterization (Figure 7a, Table 2). Through annotation analysis of these 23 candidate genes and a review of the existing literature, we identified three genes (*Sme2.5_07736.1_g00001.1*, *Sme2.5_16003.1_g00001.1*, and *Sme2.5_00423.1_g00003.1*) that are potentially closely associated with male sterility. All three are implicated in redox-related processes. Further functional prediction of homologous genes revealed that one of them, *Sme2.5_16003.1_g00001.1 (SmHTH)*, encodes a Glucose-methanol-choline (GMC) oxidoreductase family protein, which catalyzes ω-hydroxy fatty acid to ω-oxo fatty acid in the cutin, suberine, and wax biosynthesis pathway. Phylogenetic analysis indicated this gene is conserved in Solanaceae and is a homolog of the rice *HOTHEAD* (*HTH*) gene, whose knockout causes male sterility [23]. Therefore, we propose *SmHTH* as a candidate gene for rTGMS in 05ms. As this candidate’s expression did not show temperature responsiveness, but its potential functional homologs might, we identified three homologous genes (Figure 7b). All showed higher expression at high temperature, especially *Sme2.5_12650.1_g00001.1*, which also encodes a GMC oxidoreductase family protein, which was barely expressed at low temperature but highly expressed at high temperature (Figure 7c). Thus, *SmHTH* is a strong rTGMS candidate, with its constitutive low expression in 05ms potentially compensated for by induced expression of homologs (particularly *Sme2.5_12650.1_g00001.1*) under high temperature.

To identify potential regulators of the homologs’ temperature response, we examined their co-expression with TFs in the blue module. Even with a high weight threshold (0.3), 17 TFs co-expressed with *Sme2.5_12650.1_g00001.1*, including three directly linked (Figure 7d). These TFs (LOB, GeBP, C2H2 families) exhibited expression patterns similar to *Sme2.5_12650.1_g00001.1* (Figure 7e), suggesting they may regulate its temperature-responsive expression.

Notably, alignment of the 23 candidate genes with the hub genes of the six WGCNA modules showed that gene 10056 is a member of the Cyan module, a module linked to genotype. This gene was previously designated as a key candidate in our prior research [18], thereby providing additional support for its biological importance.

## 3. Discussion

TGMS lines are vital for two-line hybrid systems. Elucidating their molecular mechanisms is crucial for breeding applications. This study builds upon our previous transcriptomic work on the male sterile line 05ms [18]. While that initial study provided valuable preliminary insights, its analysis lacked a sample from a critical stage of fertile line S63. To obtain a more comprehensive and unbiased panorama of gene expression, we supplemented the dataset with samples from this key period in the current study, constructing an expanded dataset. We integrated multi-environment RNA-seq and BSA-seq data from eggplant rTGMS line 05ms and its fertile ancestor S63 to construct co-expression networks. We identified a candidate gene, *SmHTH*, highlighted key pathways (carbohydrate lipid metabolism, lipid metabolism, mRNA surveillance), defined hub genes within fertility-associated modules, and discovered TFs potentially linking temperature sensing to fertility genes via co-expression analysis, proposing a regulatory network for fertility conversion.

### 3.1. SmHTH as a Candidate Gene for Fertility Conversion

Many cloned EGMS genes are not environmentally sensitive but affect temperature-responsive targets or homologs [16]. As 05ms originated from the spontaneous mutation of S63, epigenetic variation affecting gene expression could underlie the phenotype [24]. Our integrated analysis identified 23 genes in divergent genomic regions with stable expression differences. *SmHTH* was consistently down-regulated in 05ms. It encodes a GMC oxidoreductase involved in cutin/suberine/wax biosynthesis, a homolog of rice *HTH* [23], whose disruption causes sterility. Notably, three homologs of *SmHTH* exhibited temperature-induced expression, especially *Sme2.5_12650.1_g00001.1*. It has been established that in Alfalfa (*MsNP1*) and maize (*IPE1*), genes within the GMC oxidoreductase superfamily are sufficient to induce a male sterility phenotype when knocked out in a homozygous state, with no pleiotropic effects on other characteristics [25,26]. Significantly, the biosynthesis pathways mediated by GMC oxidoreductase were also prominently enriched in the royal blue (fertility conversion) modules identified by our WGCNA. This functional convergence between the candidate gene and the co-expression network underscores a coherent biological narrative. Thus, we propose that constitutive low expression of *SmHTH* causes sterility in 05ms, compensated under high temperature by induced expression of its homologs, particularly *Sme2.5_12650.1_g00001.1*, restoring fertility.

### 3.2. Role of Carbohydrate Metabolism

KEGG analysis highlighted carbohydrate metabolism pathways (“Glyoxylate/dicarboxylate metabolism”, “Glycolysis/Gluconeogenesis”, “Fructose/mannose metabolism”, “Amino/nucleotide sugar metabolism”) in fertility-related modules. Carbohydrate metabolism is implicated in male sterility in crops like Brassica napus [27], rice [28], and wheat [29]. Nearly half the hub genes in the royal blue module were involved in sugar transport, transferase, or hydrolase activities, influencing sugar distribution and metabolism, potentially directly affecting anther development or regulating other genes via their central network positions.

### 3.3. Role of Lipid Metabolism

Lipid metabolism pathways (“Glycerolipid metabolism”, “Fatty acid metabolism”, “Fatty acid degradation”, “Fatty acid biosynthesis”, “Cutin, suberine and wax biosynthesis”) were enriched. Lipids are critical for male fertility, partly regulating anther cell expansion [30]. Many cloned sterility genes are lipid-related [31]. The candidate gene itself functions in fatty acid derivative synthesis, underscoring the potential centrality of lipid metabolism in the fertility conversion of 05ms.

### 3.4. Role of mRNA Surveillance Pathway

Enrichment of the “mRNA surveillance pathway” (detection and degradation of abnormal mRNAs) is a novel finding in this context. This quality control mechanism is essential for cellular function [32]. Hub genes in the dark red module participated in nucleotide/RNA processing, ensuring transcriptional/translational fidelity. We speculate that the accumulation of aberrant mRNAs at low temperature might contribute to sterility in 05ms.

### 3.5. TFs Link Temperature Response to Fertility Conversion

The blue module (temperature response) was enriched for TFs, suggesting they initiate the transcriptional cascade responding to temperature change [33]. TFs can integrate environmental cues with anther development by modulating lipid metabolism-related male sterility genes [34]. In our study, co-expression analysis identified three TFs (LOB, GeBP, C2H2) potentially regulating the key temperature-responsive homolog *Sme2.5_12650.1_g00001.1*. These TFs may transduce the high-temperature signal to activate the compensatory homolog.

### 3.6. Proposed Compensatory Regulatory Network

**Figure 8:**
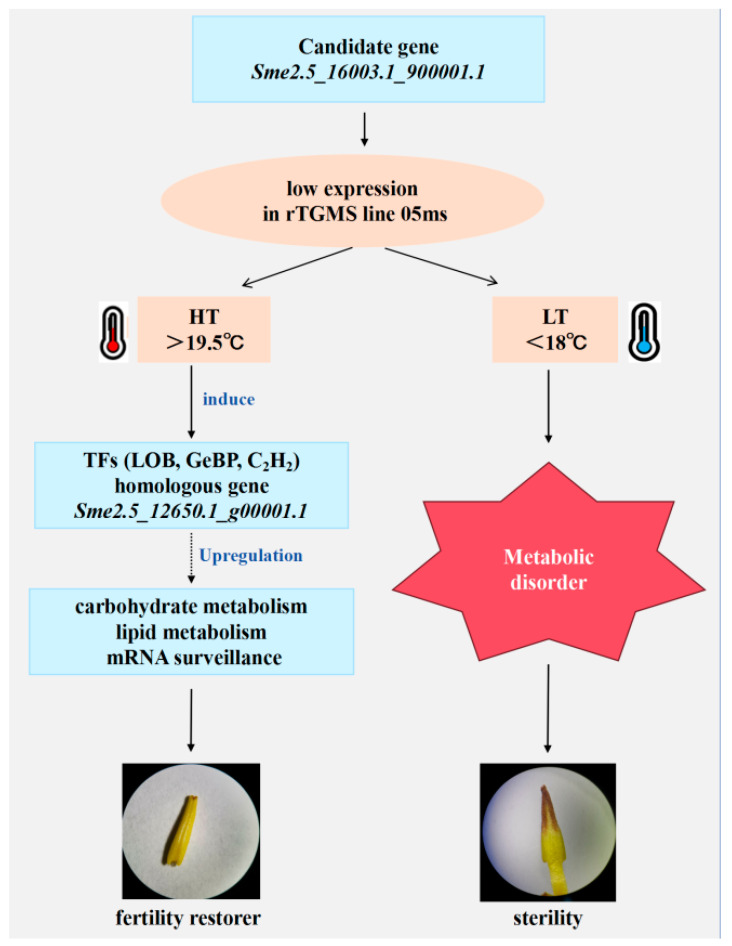
The model of thermosensitive sterility mechanism in 05ms. The reduced expression of *Sme2.5_16003.1_g00001.1* at 05ms and low temperatures led to metabolic disruptions, culminating in a sterility phenotype. Conversely, elevated expression of homolog genes and TFs at higher temperatures served to complement the role of *Sme2.5_16003.1_g00001.1*, involving sugar-related processes, fatty acid metabolism, and nucleotide processing, consequently reinstating fertility.

The eggplant rTGMS line 05ms exhibits strict temperature-dependent sterility, being infertile below 18 °C. This study utilized buds from 05ms and the fertile line S63 collected under low and high temperatures across seasons. We conducted RNA-seq under multiple environments and BSA-seq to identify key regulators and elucidate the mechanism of fertility conversion in 05ms. Co-expression networks revealed modules and hub genes related to temperature response and fertility conversion. A candidate gene for rTGMS was predicted, and a mechanism for temperature-dependent fertility conversion was proposed. Based on our findings, we propose a compensatory model for fertility conversion in eggplant rTGMS (Figure 8). At low temperature, low expression of *SmHTH* and its homologs in 05ms disrupts carbohydrate/lipid metabolism and mRNA surveillance, causing sterility. At high temperature, specific TFs (e.g., LOB, GeBP, C2H2) are induced, activating expression of the homolog *Sme2.5_12650.1_g00001.1*, which compensates for the low candidate gene expression, restoring normal metabolic/surveillance functions and fertility.

## 4. Materials and Methods

### 4.1. Plant Materials, Growth Conditions, and Sample Collection

The eggplant inbred lines used were the rTGMS line 05ms (Figure 1a and Figure S7A) and the fertile line S63 (Figure 1b and Figure S7B). The F_1_ population was produced by crossing the sterile line 05ms (P_1_) with the fertile line S63 (P_2_). F_2_ seeds were then generated by self-pollinating the F_1_ hybrids. The parental lines were cultivated in a greenhouse in Shijiazhuang (38.03° N, 114.26° E), Hebei Province, China, on 3 January 2016 and 2019 for RNA-seq. On 23 March 2016, the parental lines and 270 F_2_ individuals were transplanted into a plastic greenhouse and managed conventionally. These materials were used for BSA-seq. Line 05ms is male sterile at low temperature (<18 °C) and fertile at high temperature (>19.5 °C). Floral buds at the anther meiosis stage from seven samples (Table 1) were collected. Three biological replicates were obtained. Sepals were removed, and samples were immediately frozen in liquid nitrogen and stored at −80 °C for RNA extraction. Based on fertility phenotypic identification of the F_2_ segregating population, 20 extremely sterile and 20 extremely fertile individuals were selected. Leaf samples were collected for DNA extraction.

### 4.2. RNA Extraction, Sequencing, and Data Analysis

Total RNA was extracted using the DP441 Kit (TIANGEN, Beijing, China). RNA sequencing was performed by Novogene Bioinformatics Technology Co., Ltd. (Beijing, China) on an Illumina HiSeq 4000 platform. Data analysis followed previously described methods [18]. The reference genome sequence and annotation (SME_r2.5.1) were obtained from the Eggplant Genome Database (ftp://ftp.kazusa.or.jp/pub/eggplant/ (accessed on 19 June 2019)). Clean data from this study and previous work were aligned to the reference genome using Hisat2 (2.0.5). Gene expression levels were quantified as FPKM (Fragments Per Kilobase Million). Differential expression analysis was conducted using the DESeq R package (v1.18.0). Genes with an adjusted *p*-value < 0.05 were considered differentially expressed genes (DEGs). Gene Ontology (GO) enrichment and Kyoto Encyclopedia of Genes and Genomes (KEGG) pathway analyses for DEGs or module genes were performed using ClusterProfiler (3.4.4).

### 4.3. Weighted Gene Co-Expression Network Analysis (WGCNA)

WGCNA was performed using the WGCNA R package v 1.68. Genes with expression levels (FPKM) in the top 25th percentile based on standard deviation were included. Hub genes within each module were defined as those with connectivity in the top 10th percentile, as described by previous reports [35,36,37].

### 4.4. DNA Extraction, Sequencing, and Data Analysis

Genomic DNA was extracted using the DE-06112 Kit (FOREGENE, Chengdu, China). A bulk segregant pool of the 05ms lines (S pool) was prepared by pooling equal amounts of DNA from 20 male sterile individuals. Similarly, a bulk pool of S63 lines (F pool) was constructed by combining DNA from 20 male fertile individuals in equal proportions. Following quality assessment of the DNA from 05ms, S63, S pool, and F pool, whole-genome resequencing was conducted by Novogene Bioinformatics Technology Co., Ltd. (Beijing, China) on an Illumina HiSeq 4000 platform. The resequencing depths for the parental lines and the two bulked pools were 10× and 20×, respectively. Raw reads were subjected to quality control, and the resulting high-quality reads from all four pools were aligned to the eggplant reference genome (SME_r2.5.1) using BWA software (version 0.7.10). Sequence Alignment/Map (SAM) files were converted to Binary Alignment/Map (BAM) format using SAMtools (version 0.1.19). PCR duplicates were removed from the alignment files using the “rmdup” command in SAMtools. SNP and InDel markers were identified across multiple samples using the UnifiedGenotyper function in the Genome Analysis Toolkit (GATK v3.3). Functional annotation of SNPs and InDels was performed with ANNOVAR v2013Aug23, based on the reference genome GFF3 annotation file.

### 4.5. Correlation Region Analysis of BSA

Candidate genomic regions associated with the trait of interest were identified by assessing differences in allele frequency between the bulked pools via calculation of the SNP index. Specifically, the Δ(SNP index) was computed using a sliding window approach with a window size of 1 Mb and a step size of 1 kb. We performed trait-linked region mapping based on SNP and InDel markers, designating windows exceeding the 95% confidence threshold as candidate intervals.

### 4.6. Combined Analysis of BSA-Seq and RNA-Seq

Gene sets corresponding to the BSA-seq candidate intervals were first identified, and their expression data (FPKM) were retrieved from the RNA-seq dataset. K-means clustering (k = 6) was then performed to group genes by expression patterns linked to fertility, followed by the construction of a heatmap to visualize these patterns across samples, utilizing the Novogene online bioinformatics platform for both analyses.

### 4.7. Data Visualization

Networks were visualized using Cytoscape (v3.7.0). Venn diagrams, heatmaps, and dot plots were generated using the VennDiagram v 1.6.20, pheatmap v 1.0.12, and ggplot2 v 3.1.1 R packages, respectively.

### 4.8. qRT-PCR Validation of Hub Genes

Total RNA was extracted using the DP441 Kit (TIANGEN, Beijing, China) and the quality was checked by agarose (CA1341, Beijing Coolaber Science & Technology Co., Ltd., Beijing, China) gel electrophoresis. cDNA was synthesized using a Reverse Transcription Kit (JKR23014, GeneCreate, Wuhan, China) and used as the template for quantitative real-time PCR (qRT-PCR) analysis. Eight hub genes were validated by quantitative real-time PCR (qRT-PCR) using an ABI 7500 Real-Time PCR System (Applied Biosystems, Waltham MA, USA), using the 2 × SYBR Green qPCR Mix (AH0101-A, Shandong Sparkjade Biotechnology Co., Ltd., Jinan, China). Gene-specific primers are synthesized by GenScript Corporation (Nanjing, China); sequences are listed in Table S8.

### 4.9. Statistical Analysis

Data are presented as means ± standard error (SE) from multiple independent experiments. qPCR data were analyzed using the 2^−ΔΔCt^ method. Differences between means were assessed using Duncan’s multiple range test, with significance set at *p* < 0.05 or *p* < 0.01.

## 5. Conclusions

Through a systems biology approach centered on WGCNA, integrated with BSA-seq, we identified key biological processes and candidate gene *SmHTH* for rTGMS in eggplant. We propose a compensatory model where high-temperature-induced expression of functional homologs restores fertility. This study provides a foundational model and genetic resources for advancing two-line hybrid breeding in eggplant.

## Figures and Tables

**Table 1:** The growth conditions and fertility characteristics of 05ms and S63 in eggplant (*Solanum melongena* L.).

Material	Code	Fertility	Low Temperature (°C)	High Temperature (°C)	Date (Month)	Data Source
05ms	MLa	sterile	15–18	<35	4–6	Li et al., 2019 [18]
	MLb	sterile	15–18	<35	4–6	New samples in 2019
	MH	fertile	>20	<35	7–9	Li et al., 2019 [18]
	MZ	sterile	10–18	<35	10–11	Li et al., 2019 [18]
S63	CL	fertile	15–18	<35	4–6	New samples in 2019
	CH	fertile	>20	<35	7–9	Li et al., 2019 [18]
	CZ	fertile	10–18	<35	10–11	Li et al., 2019 [18]

**Table 2:** Functional analysis of 23 candidate genes screened from BSA-seq intervals based on expression level.

Gene ID	Sly	Tair	Swissprot
*Sme2.5_07736.1_g00001.1*	Solyc08g062970.1.1	AT4G33040.1	GRXC6_ARATH Glutaredoxin-C6
*Sme2.5_00021.1_g00023.1*	Solyc06g076620.2.1	AT5G52370.1	No hit
*Sme2.5_00018.1_g00018.1*	Solyc06g068240.2.1	AT1G15690.1	AVP1_ARATH Pyrophosphate-energized vacuolar membrane proton pump 1
*Sme2.5_00182.1_g00005.1*	Solyc08g065420.2.1	AT4G32980.1	ATH1_ARATH Homeobox protein ATH1
***Sme2.5_10056.1_g00002.1***	**Solyc06g008320.2.1**	**AT4G14340.1**	**KC1D_ARATH Casein kinase I isoform delta-like**
*Sme2.5_00656.1_g00012.1*	Solyc11g006420.1.1	No hit	No hit
*Sme2.5_00339.1_g00008.1*	Solyc12g009540.1.1	AT4G23160.1	COPIA_DROME Copia protein
*Sme2.5_00008.1_g00035.1*	Solyc06g068890.1.1	No hit	M1CT33_SOLTU Uncharacterized protein
***Sme2.5_16003.1_g00001.1***	**Solyc06g062600.2.1**	**AT1G72970.1**	**HTH_ARATH Protein HOTHEAD**
*Sme2.5_02187.1_g00009.1*	Solyc02g078130.2.1	AT4G20970.1	K4B938_SOLLC Uncharacterized protein
*Sme2.5_00423.1_g00001.1*	Solyc07g055160.2.1	AT2G42690.1	M1BTN2_SOLTU Uncharacterized protein
*Sme2.5_00423.1_g00003.1*	Solyc07g055190.2.1	AT5G05340.1	PER52_ARATH Peroxidase 52
*Sme2.5_00339.1_g00011.1*	Solyc02g085760.2.1	AT5G38510.2	M1AYG6_SOLTU Uncharacterized protein
*Sme2.5_00656.1_g00001.1*	Solyc11g006230.1.1	AT5G28640.1	M0ZKB5_SOLTU Uncharacterized protein
*Sme2.5_00281.1_g00003.1*	Solyc01g104470.2.1	AT1G25260.1	M1C6X3_SOLTU Uncharacterized protein
*Sme2.5_00844.1_g00009.1*	Solyc11g051150.1.1	AT5G65070.3	POLX_TOBAC Retrovirus-related Pol polyprotein from transposon TNT 1–94
*Sme2.5_03276.1_g00004.1*	Solyc11g020990.1.1	No hit	IP22_SOLLC Proteinase inhibitor type-2 TR8
*Sme2.5_01098.1_g00005.1*	Solyc01g091230.2.1	AT2G45340.1	IMK2_ARATH Probably inactive leucine-rich repeat receptor-like protein kinase
*Sme2.5_00481.1_g00009.1*	-	-	-
*Sme2.5_03554.1_g00006.1*	Solyc06g009650.2.1	AT4G02030.2	VPS51_DICDI Vacuolar protein sorting-associated protein 51 homolog
*Sme2.5_02419.1_g00009.1*	Solyc12g056100.1.1	AT1G64230.2	UBC28_ARATH Ubiquitin-conjugating enzyme E2 28
*Sme2.5_00281.1_g00016.1*	Solyc01g104630.2.1	AT2G06000.2	M1CWY2_SOLTU Uncharacterized protein
*Sme2.5_01192.1_g00007.1*	Solyc09g092690.2.1	AT3G25230.1	FKB62_ARATH Peptidyl-prolyl cis-trans isomerase FKBP62

Note: The bold values were indicated important key genes.

## Data Availability

The data used to support the findings of this study are available upon request from the corresponding author.

## Supplementary Materials

The following supporting information can be downloaded at: https://www.mdpi.com/article/10.3390/ijms262210873/s1.
