# Supplementary material for: Systematic Analysis of Fertility Conversion via WGCNA Implicates a Compensatory Regulatory Network in a Reverse Thermosensitive Genic Male Sterility Line of Eggplant (Solanum melongena L.)
Source: Int J Mol Sci. 2025 Nov 9;26(22):10873. doi: 10.3390/ijms262210873 (PMC12652905; doi:10.3390/ijms262210873)
Supplement: Supplementary file 1 [file ijms-26-10873-s001.zip › Supplementary Figures.pdf]

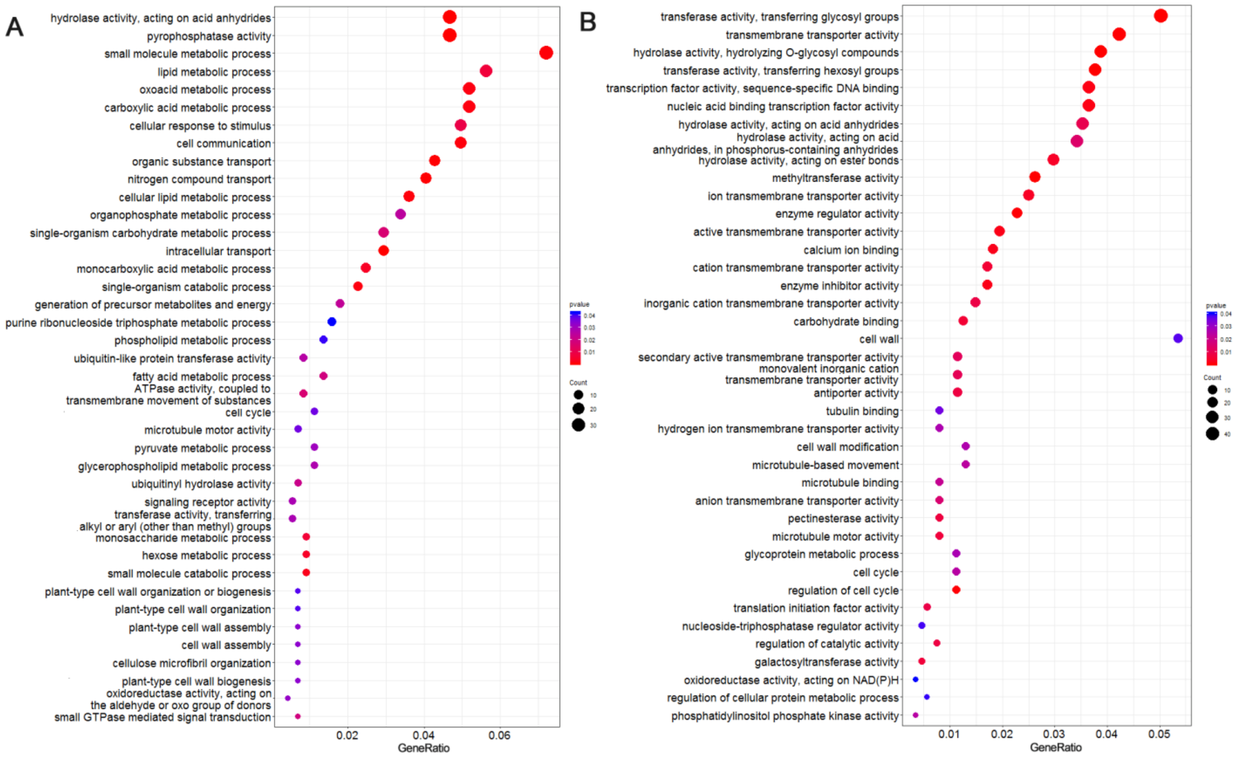

Figure S1. GO enrichment of genes in cluster I and II indicated in Figure **1b** and Figure **1c**.

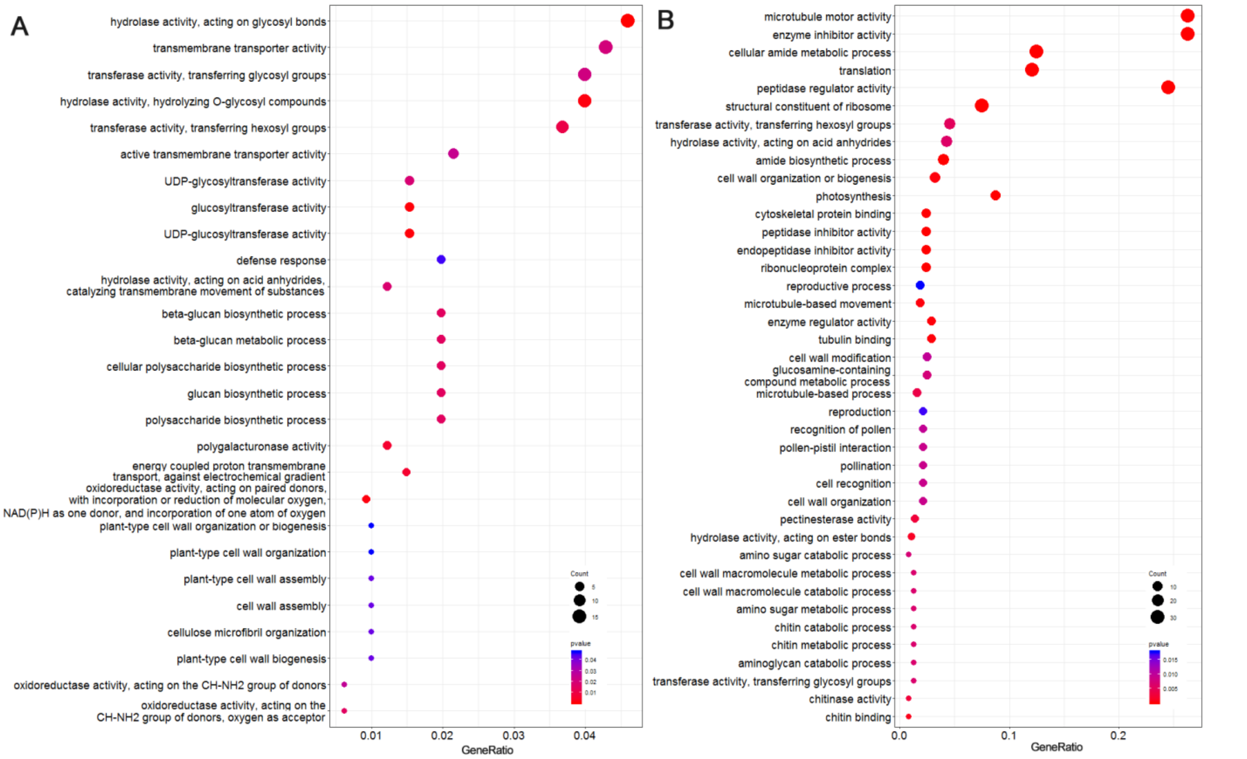

Figure S2. GO enrichment of genes in cluster III and IV indicated in Figure **1d**.

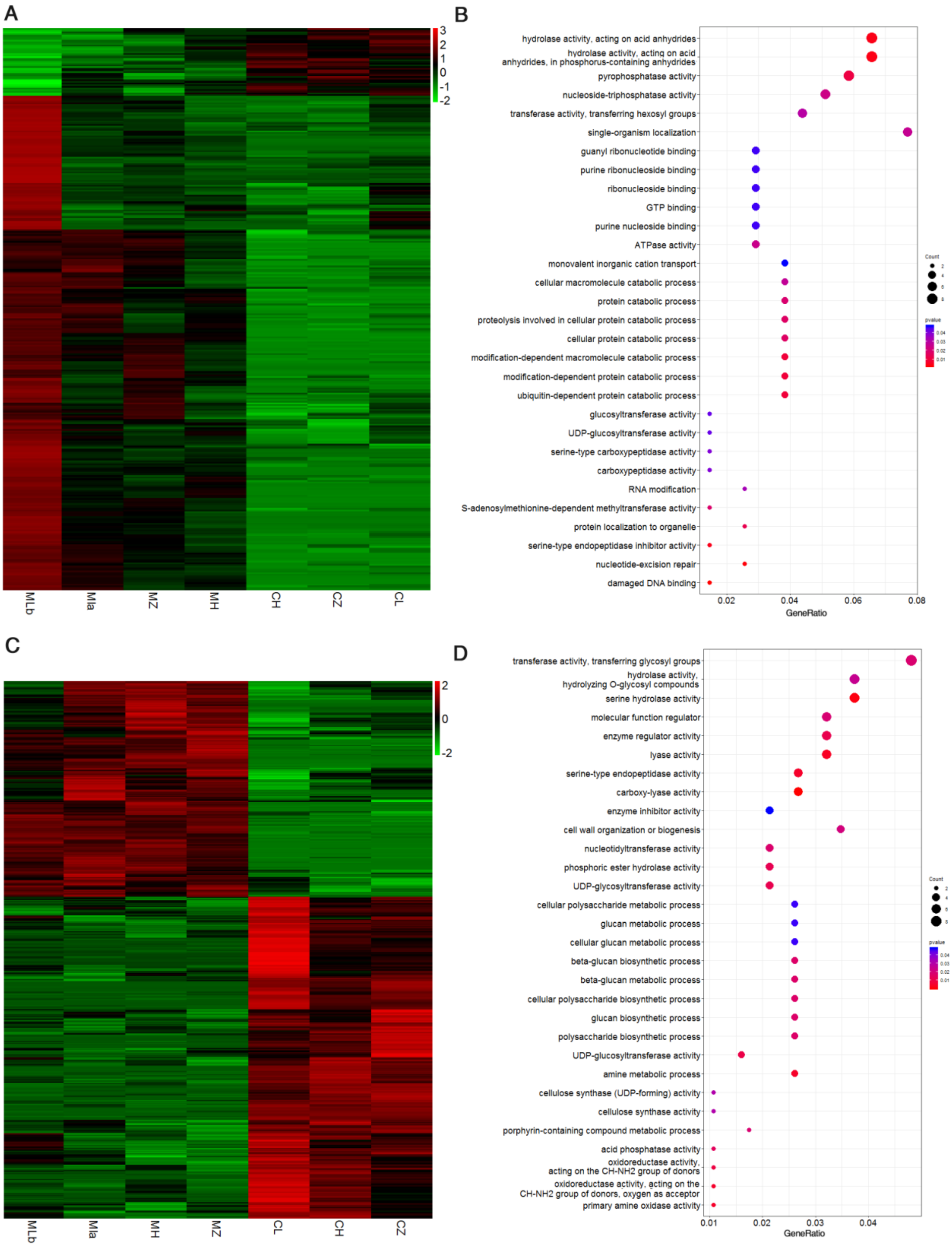

Figure S3. The expression difference and GO enrichment analysis of genes in royalblue and darkred modules. A and C, heatmap showing the expression difference of genes in royalblue (A) and darkred (C) modules in different samples. B and D, Dotplot showing the result of GO enrichment analysis of genes in royalblue (B) and darkred (D) modules.

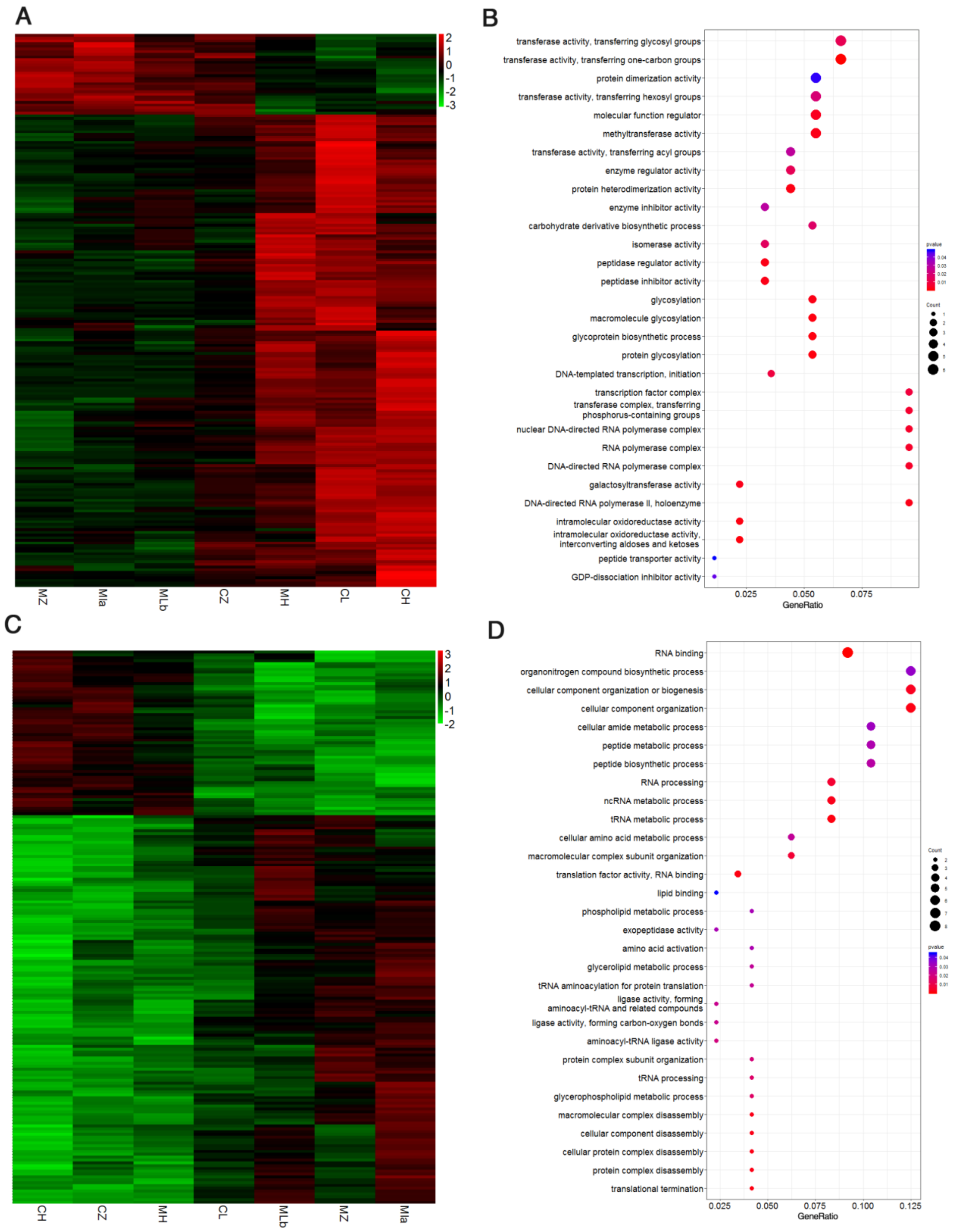

Figure S4. The expression difference and GO enrichment analysis of genes in grey60 and cyan modules. A and C, heatmap showing the expression difference of genes in grey60 (A) and cyan (C) modules in different samples. B and D, Dotplot showing the result of GO enrichment analysis of genes in grey60 (B) and cyan (D) modules.

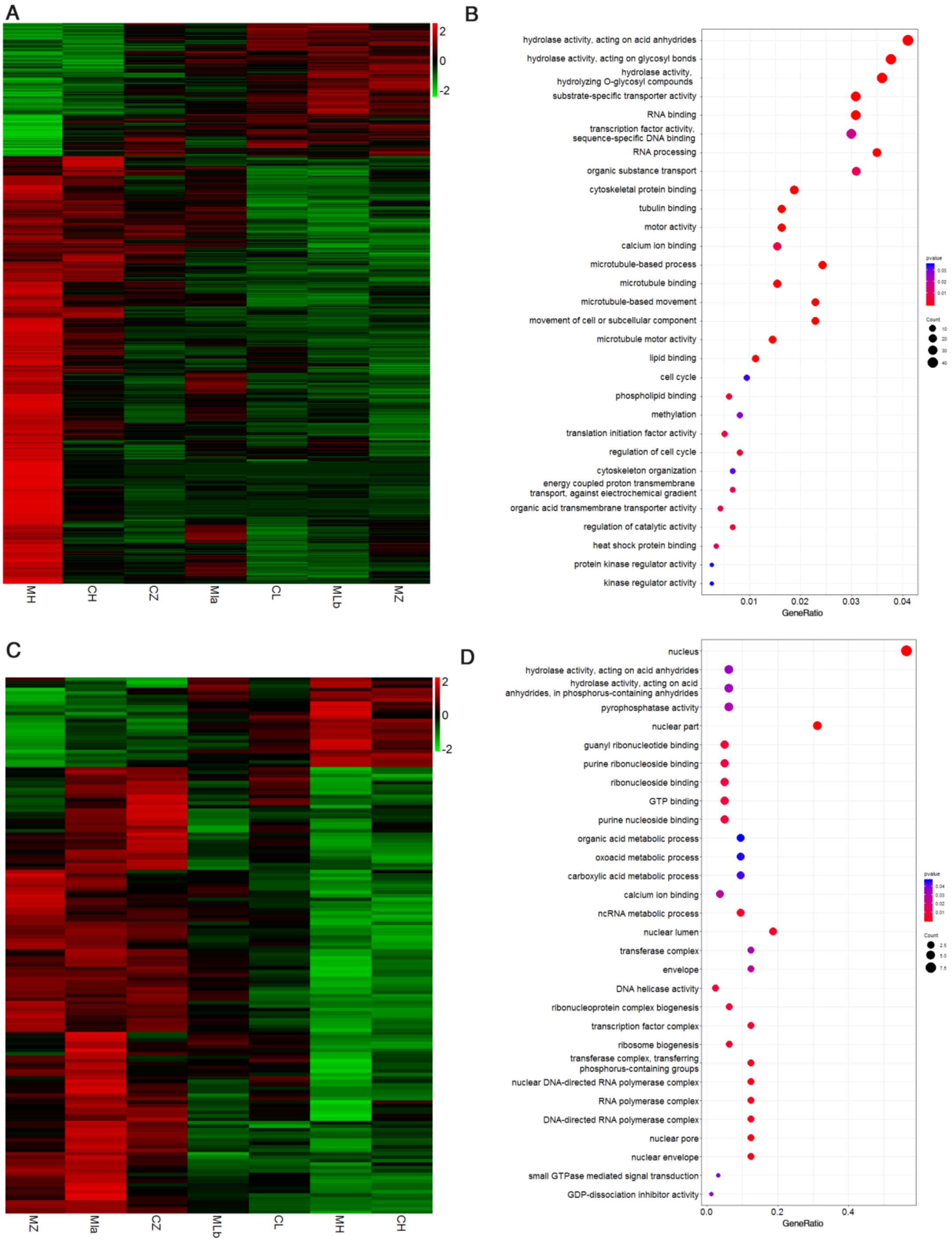

Figure S5. The expression difference and GO enrichment analysis of genes in blue and darkgreen modules. A and C, heatmap showing the expression difference of genes in blue (A) and darkgreen (C) modules in different samples. B and D, Dotplot showing the result of GO enrichment analysis of genes in blue (B) and darkgreen (D) modules.

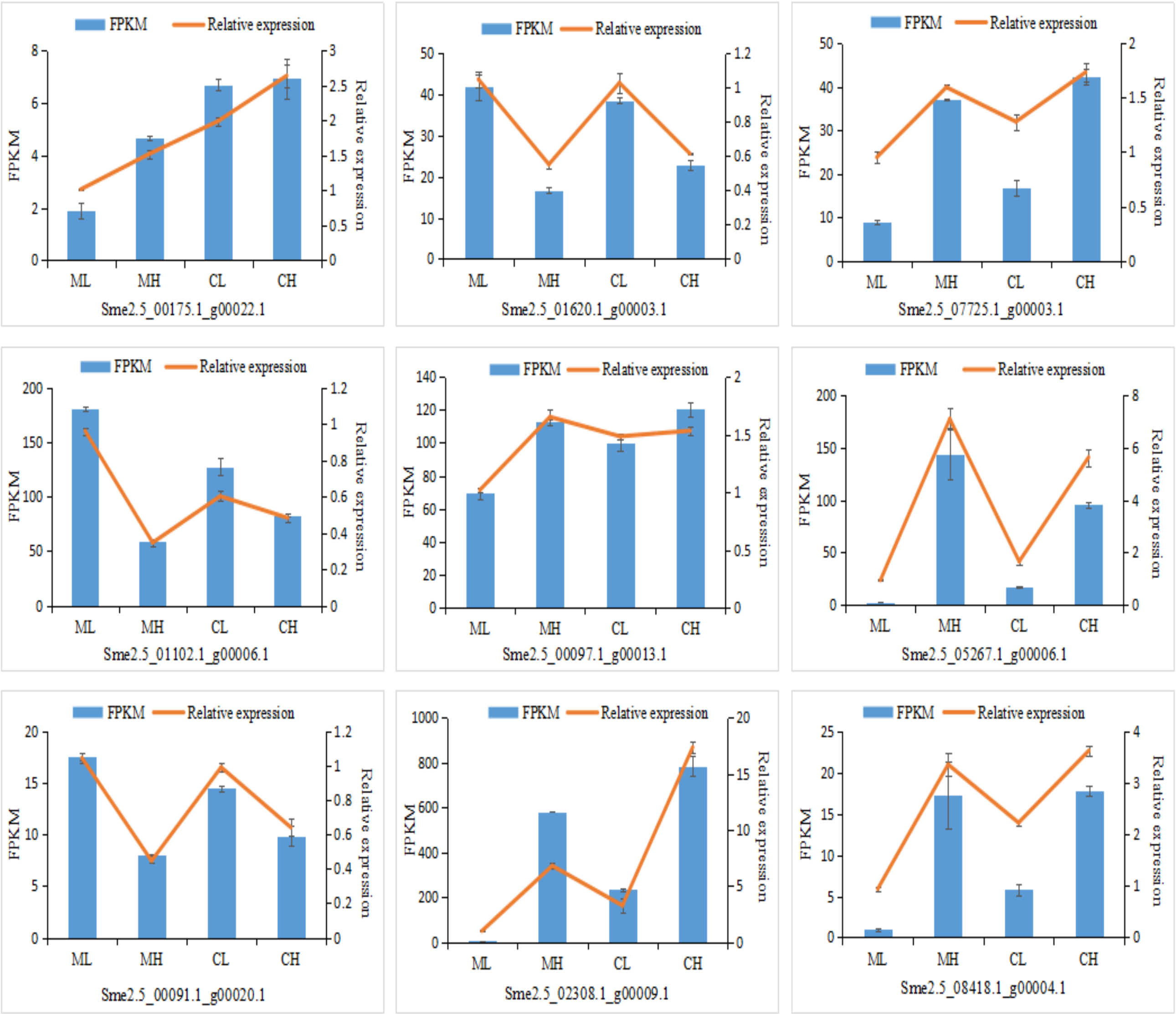

Figure S6. qPCR validation of selected genes.

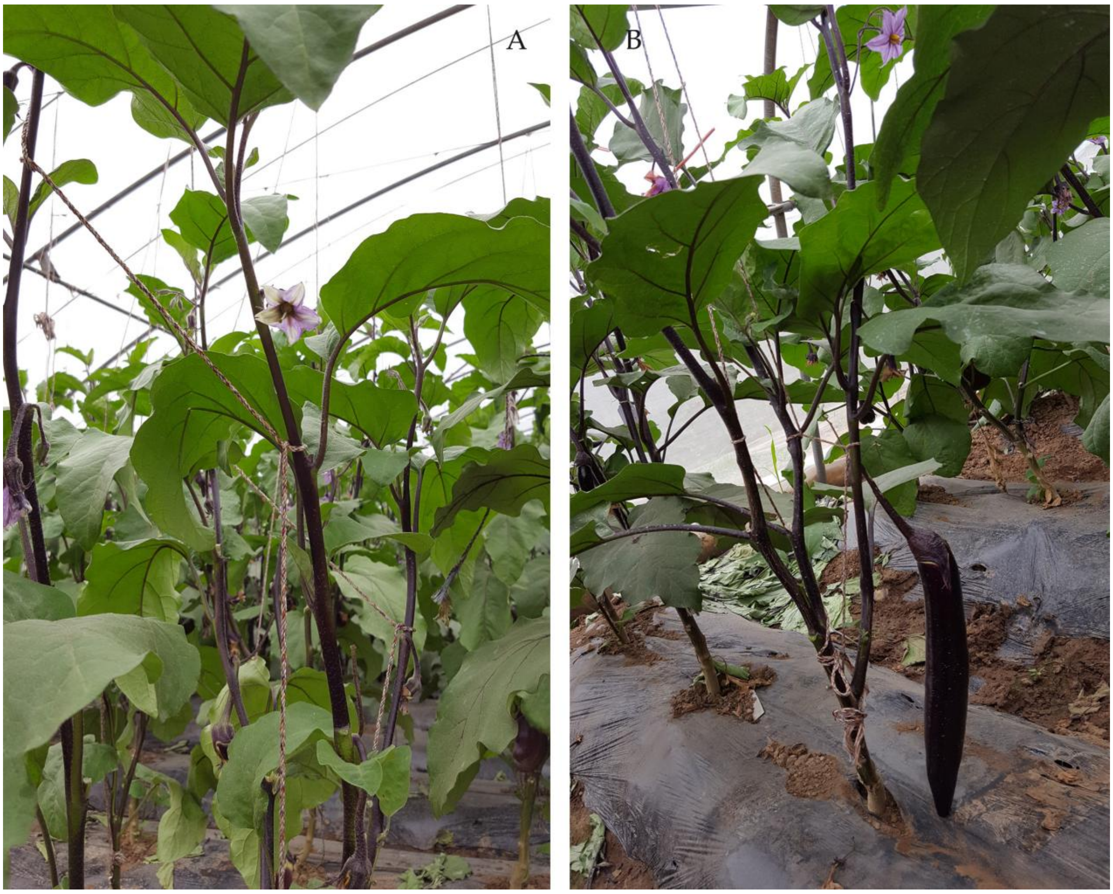

Figure S7. The plant phenotype of male sterile line 05ms and male fertile line S63. A, 05ms. B, S63.
